# On the MIAME Standards and Central Repositories of Microarray Data

**DOI:** 10.1002/cfg.238

**Published:** 2003-02

**Authors:** Steve Oliver


**Comparative and Functional Genomics**
*Comp Funct Genom* 2003; **4**: 1.
Published online in Wiley InterScience (www.interscience.wiley.com). **DOI:** 10.1002/cfg.238

**Editorial**

### On the MIAME standards and central repositories of microarray data

CFG has been approached, along with other journals publishing in the area of genomics, by the Microarray Gene Expression Database (MGED) society. We have been urged to use its recommendations when deciding whether to accept a paper for publication. As frequent readers of this journal will know, CFG supports the development and usage of standards and ontologies in genomics, as evidenced in this issue by our coverage of two key ontology meetings.

It is the decision of our Editorial Board that, from this issue onwards, compliance with the Minimum Information About a Microarray Experiment (MIAME; 1, 2) standards will be mandatory — except in cases where a microarray is used to identify one, or a few, genes, and these individual genes are then the subject of much deeper analysis that validates their selection. In this latter case, the microarray is merely a 'primary screen' and, just as for a genetic screen, one wouldn't necessarily publish all the data at once.

On the issue of data submission to central repositories (ArrayExpress and GEO), CFG prefers to be, for now, less strict. We certainly encourage authors to submit their data to a central repository, and we will eventually make such deposition mandatory. This will apply when the set-up of the central databases has matured, and deposition is a straightforward process for any user. However, from now on, authors are obliged to make sure, and confirm to us in writing, that their entire data set will be available on the Internet, without charge or limitation, for at least 2 years from the date of publication, even if their data is not deposited centrally. In cases where it is not possible for authors to host their dataset, CFG will investigate providing access to the data on its own website.

**Steve Oliver**

### References

1. Brazma A, Hingamp P, Quackenbush J, *et al*. 2001. Minimum information about a microarray experiment (MIAME)-toward standards for microarray data. *Nature Genet* **29**(4): 365–371.
2. MIAME guidelines for authors, editors and reviewers of microarray gene expression papers: **http://www.mged.org/Workgroups/MIAME/miame_checklist.html**

Copyright © 2003 John Wiley & Sons, Ltd.
